# MiR-31 suppresses lung adenocarcinoma cell proliferation through CDK1 and E2F2-mediated cell cycle arrest

**DOI:** 10.1007/s12672-025-04362-6

**Published:** 2026-01-09

**Authors:** Pan Sun, Man Zhang, Shanshan Wang, Yulin He, Chenjing Lin, Yi Tian, Jizhuang Luo, Kai Wang

**Affiliations:** 1 Central Laboratory, Shanghai Chest Hospital, Shanghai Jiao Tong University School of Medicine, Shanghai, People’s Republic of China; 2 Department of Radiology, Xiangyang Hospital of Traditional Chinese Medicine, Hubei University of Chinese Medicine, Xiangyang, People’s Republic of China; 3 Precision Medicine Research Center, Clinical Research Center, Shanghai Chest Hospital, Shanghai Jiao Tong University School of Medicine, Shanghai, People’s Republic of China; 4 Department of Chemotherapy, Fujian Geriatric Hospital, Fujian Provincial Hospital North Branch, Fuzhou, Fujian People’s Republic of China; 5 Department of Clinical Medical, Sichuan Provincial People’s Hospital East Sichuan Hospital & Dazhou First People’s Hospital, Dazhou, People’s Republic of China; 6 Department of Thoracic Surgery, Shanghai Jiao Tong University School of Medicine, Shanghai, People’s Republic of China

**Keywords:** miR-31, CDK1, E2F2, Lung adenocarcinoma (LUAD), Cell proliferation

## Abstract

**Background:**

MicroRNAs (miRNAs) exert pivotal regulatory functions in cancer initiation, progression, and metastasis by regulating cell proliferation-cycle related genes. However, tumor-associated miRNAs in lung adenocarcinoma (LUAD) remains incompletely characterized.

**Results and findings:**

By interrogating TCGA mRNA-Seq datasets, we identified 1672 differentially expressed genes (DEGs) implicated in proliferation-cycle regulation in LUAD. A significant overrepresentation of transmembrane signal receptors, kinases, and TFs was observed among the DEGs, with primary enrichment in signaling pathways such as chemokine/cytokine, Wnt, EGF, Cadherin, and p53 cascades. Remarkably, *CDK1* and *E2F2* were characterized as key proliferation-cycle regulatory genes, demonstrating > fivefold transcriptional up-regulation in LUAD specimens compared to normal lung tissues (p < 0.001). Mechanistically, pharmacological CDK1 inhibition using fostamatinib or alsterpaullone reversed aberrant proliferative phenotypes in LUAD cells, demonstrating therapeutic reversibility in vitro. Concurrently, DEmiRNA and target analysis identified miR-31 as a critical regulator of CDK1/E2F2, showing elevated expression in LUAD.

**Clinical implications:**

Collectively, our study establishes miR-31 as a novel biomarker for LUAD proliferative potential and implicates the miR-31/CDK1-E2F2 network as a promising target for disrupting LUAD progression. These findings establish a miRNA-centric precision therapeutic paradigm for effectively suppressing oncogenic proliferation in LUAD.

**Supplementary Information:**

The online version contains supplementary material available at 10.1007/s12672-025-04362-6.

## Introduction

LUAD, the most common histological subtype of lung cancer, remains a leading cause of cancer-related deaths globally [1, 2]. Despite advancements in molecular diagnostics and targeted therapies, the 5-year survival rate remains under 20% due to high recurrence rates and metastatic potential, both closely linked to aberrant cell proliferation [2, 3]. This underscores the critical need for identifying robust biomarkers that can predict proliferative phenotypes and guide precision therapeutic strategies.

Emerging evidence highlights tumor-associated miRNAs as key regulators of tumor progression, with dysregulated expression directly impacting cell cycle machinery and proliferation pathways [4, 5]. For instance, miR-30 family members (miR-30a/c) exhibit tumor-suppressive properties by modulating cell cycle checkpoints [6]. while miR-1293 and miR-873 promote aggressive phenotypes through proliferation-related targets [7, 8]. Clinical studies support the diagnostic/prognostic utility of serum miRNAs like miR-142, miR-409, miR-223, and miR-146a [9], emphasizing the translational potential of miRNA biomarkers in LUAD management. Despite recent advances, more tumor-suppressive miRNAs remain underexplored, and their regulatory roles in LUAD proliferation remain incompletely characterized, warranting further mechanistic investigations to unlock their therapeutic potential.

This study aimed to address this gap by integrating multi-omics analyses to identify: (1) proliferation-cell related Differential Expressed Genes (DEGs) in LUAD vs. normal tissues, (2) candidate tumor-suppressive miRNAs regulating these DEGs, and (3) miRNA-mRNA regulatory interactions associated with prognostic signatures. Through systematic validation via survival analysis and functional assays, we sought to establish a miRNA-centered precision medicine framework for counteracting LUAD proliferation.

## Materials and methods

### Data acquisition, preprocessing and differential expression analysis

RNA-Seq raw read counts for LUAD were retrieved from The Cancer Genome Atlas (TCGA) [10], comprising 535 tumor samples and 59 solid tissue normal samples for RNA-Seq and 521 tumor samples and 46 solid tissue normal samples for miRNA-Seq. Non-coding transcripts were excluded, retaining protein-coding genes for downstream analysis. DESeq2 (v1.38.3) [11] was implemented to identify DEGs and differentially expressed miRNAs (DEmiRNAs). A fold change (FC) ≥ 1.5 and an adjusted p-value < 0.05 were adopted to filter out insignificant DEGs/DEmiRNAs. These thresholds are widely used in literatures to balance sensitivity/specificity, ensuring robust detection of biologically relevant changes while reducing false positives.

### Functional enrichment analysis and classification

The Gene Ontology (GO) and KEGG pathway enrichment of DEGs were analyzed by the R package clusterProfiler (v4.6.2). The PANTHER webserver (v19.0) [12] was utilized to categorize proliferation-cycle associated DEGs into protein classes and pathways using Homo sapiens reference database. Besides, Metascape-based (v3.5.20250101) [13] pathway and process enrichment analysis were performed using a p-value cutoff of 0.01 and minimum enrichment threshold of 1.5. Top 20 clusters ranked by enrichment significance were visualized with color-coded networks representing distinct biological modules.

### Additional RNA-Seq validation experiment

External validation of gene expression was carried out using two independent LUAD datasets, GSE40419 [14] and GSE140343 [15], from the NCBI Gene Expression Omnibus (GEO). The GSE40419 dataset comprises 90 tumor tissues and 77 adjacent normal tissues, whereas the GSE140343 dataset includes 51 tumor tissues and 49 adjacent normal counterparts. The method for DEG identification is identical to the one applied to the above mentioned TCGA dataset.

### OncomiR analysis

OncomiR [16] was utilized to identify dysregulated miRNAs implicated in LUAD tumorigenesis. In this study, tumor-associated miRNAs with LUAD were retrieved via clicking “Tumor Development” under “Search by Cancer Type” (Student’s paired t-test, p ≤ 0.05). The corresponding “stage and grade” and “survival outcome analysis” were performed for the selected miRNAs. survival outcomes were analyzed using univariate Cox regression (log-rank p ≤ 0.05) to link miRNA expression with clinical prognosis.

### MiR-31 target identification

miR-31 targets were predicted using miRWalk2.0 [17], a comprehensive database integrating 13 prediction algorithms and experimental validations. All miRNA-mRNA interactions were meticulously selected based on a set of well-defined criteria to ensure the reliability and biological relevance of the predicted interactions. Specifically, the following parameters were employed: (1) The maximum free energy of the miRNA-mRNA duplex formation was set at − 15.0 kcal/mol to ensure a stable yet specific interaction. (2) The miRNA-mRNA binding region needs to be at least 10 base pairs (bp) long for effective regulation. (3) The binding was required to occur within the 3' untranslated region (3' UTR) of the mRNA. (4) An extensive literature review was conducted to search for experimental validations of miR-31—mRNA target pairs. (5) The mRNA targets were confined to DEGs that were implicated in cell proliferation and cell cycle regulation. Following the filtration process described above, the mRNA target pairs were prioritized to facilitate the establishment of a miRNA mediated interaction network.

### Protein–protein interaction network construction

High-confidence PPI data were aggregated from BioGRID (Release 4.4.231) [18], IntAct (Release 2024–11–29) [19], and STRING (v12.0, score ≥ 0.9) [20] database. Interactions supported by ≥ 2 experimental methods or literature sources were retained to minimize false positives. This conservative approach minimizes cumulative error propagation when integrating multi-database evidence, ensuring comprehensive coverage of the integrated PPI network and robust network analysis while acknowledging potential sensitivity trade-offs. Network visualization and topological analysis were performed using Cytoscape 3.8.0 [21].

### Survival analysis

Overall survival of LUAD patients was analyzed by Gene Expression Profiling Interactive Analysis (GEPIA2) [22], and patients were stratified into high- and low-expression groups based on the 25th and 50th percentile cutoff of gene expression levels. The prognostic significance of gene expression was evaluated using Cox proportional hazards regression models, with hazard ratios (HRs) and 95% confidence intervals (CIs) calculated for each group comparison. Additionally, Kaplan–Meier survival curves were plotted, and the log-rank test was performed to assess differences in survival between groups. A p-value < 0.05 was considered statistically significant.

### Drug discovery

The Connectivity Map (v1.1.1.43) [23] analysis was conducted to uncover potential therapeutic drugs that specifically target CDK1 and E2F2. Additionally, the DrugBank database (Release 5.1.13) [24] (https://go.drugbank.com/), a meticulously curated molecular repository containing comprehensive drug-target interaction data, was utilized to identify extra inhibitors for these two targets. Advanced search filters were applied to retrieve experimentally validated compounds with direct targeting evidence, ensuring therapeutic relevance to LUAD.

## Results

### DEGs between LUAD and adjacent normal lung cells

**Fig. 1:**
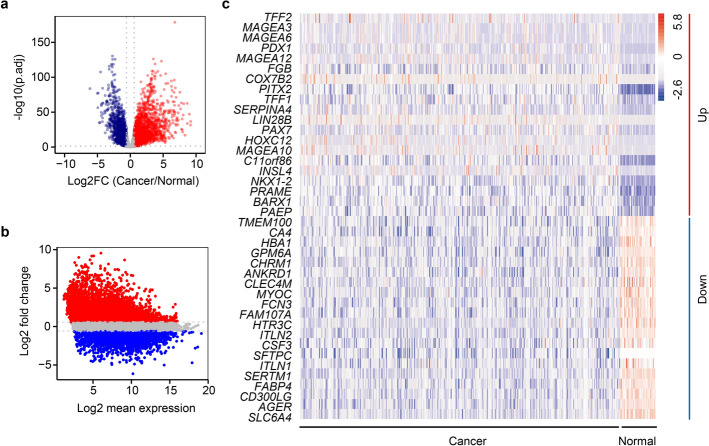
Differential gene expression profiling in LUAD vs. normal lung tissues. **a** Volcano plot visualization of transcriptome-wide expression changes. **b** MA plot distribution of differential expression patterns. **c** Heatmap of top 20 up/down-regulated genes ranked by fold change

To identify DEGs between LUAD cells and the adjacent lung normal cells, we performed transcriptome profiling of 535 LUAD tumors and 59 adjacent normal tissues using TCGA data [10]. After filtering non-coding transcripts, 19,581 protein-coding genes were analyzed, revealing 7827 DEGs with biological significance (4902 up-regulated, 2925 down-regulated) (Fig. 1a, Table S1). Notably, genes with high expression variability exhibited lower abundance (Fig. 1b), while the top 20 up- and down-regulated DEGs are ranked in Fig. 1c.

### Functional enrichment of DEGs highlights cell cycle deregulation

**Fig. 2:**
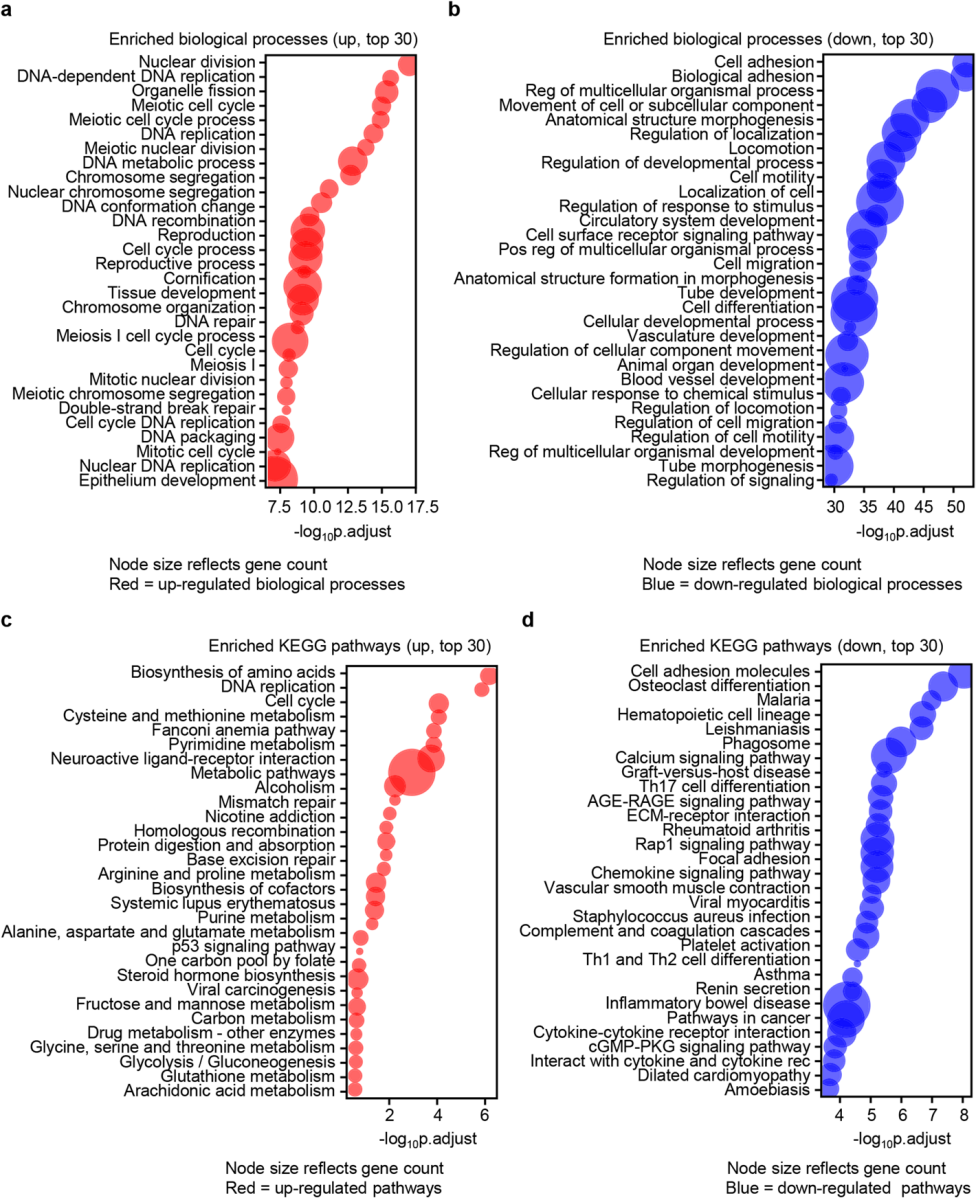
Functional enrichment analysis of DEGs in LUAD pathogenesis. **a**-**b** Bubble plot of the top 30 enriched biological processes associated with (**a**) up- and (**b**) down-regulated DEGs. **c**, **d** Bubble plot of the top 30 enriched KEGG pathways associated with (**c**) up- and (**d**) down-regulated DEGs. Node size is proportional to the number of genes assigned to each GO term or KEGG pathway

The enriched function and pathways of the perturbed DEGs were checked subsequently. GO analysis revealed striking enrichment of up-regulated DEGs in cell cycle-related processes, including nuclear division, DNA replication, and chromosome segregation (Fig. 2a). Conversely, down-regulated DEGs were primarily linked to cell adhesion and motility (Fig. 2b). From the perspective of molecular functions, up-regulated genes predominantly associated with transmembrane transport of ions and inorganic entities, as well as crucial DNA-related activities like DNA binding, catalytic actions on DNA, and ion channel regulation. In contrast, down-regulated genes primarily center on a wide array of molecular binding interactions, including those with receptors, ligands, and proteins, and are implicated in various cellular, signaling, and enzymatic activities, with a notably reduced emphasis on transport and DNA-associated processes. Additionally, up-regulated genes mainly localize to cellular components associated with chromosome-related structures, such as chromosomes and chromatin, as well as nuclear components like the nucleoplasm. They are also highly represented in complexes involved in transport processes (e.g., transmembrane transporter complexes) and DNA–protein interactions (e.g., protein-DNA complexes). while, down-regulated genes are primarily concentrated in plasma membrane-associated regions, cell projections, the extracellular matrix, and diverse vesicular and cytoskeletal structures (uploaded as related files). KEGG pathway mapping confirmed these trends, with up-regulated genes overrepresented in DNA replication and cell cycle pathways (Fig. 2c), and down-regulated genes associated with focal adhesion and cell junction dynamics (Fig. 2d). These findings are congruent with the emergence of cell cycle dysregulation as a hallmark of LUAD pathogenesis, underscoring its pivotal role in driving neoplastic progression [5]. The enrichment of cell adhesion related biological processes and pathways in down-regulated genes further implies heightened LUAD cellular motility, a critical determinant of metastatic potential.

### Proliferation-cycle associated DEGs and functional implications

**Fig. 3:**
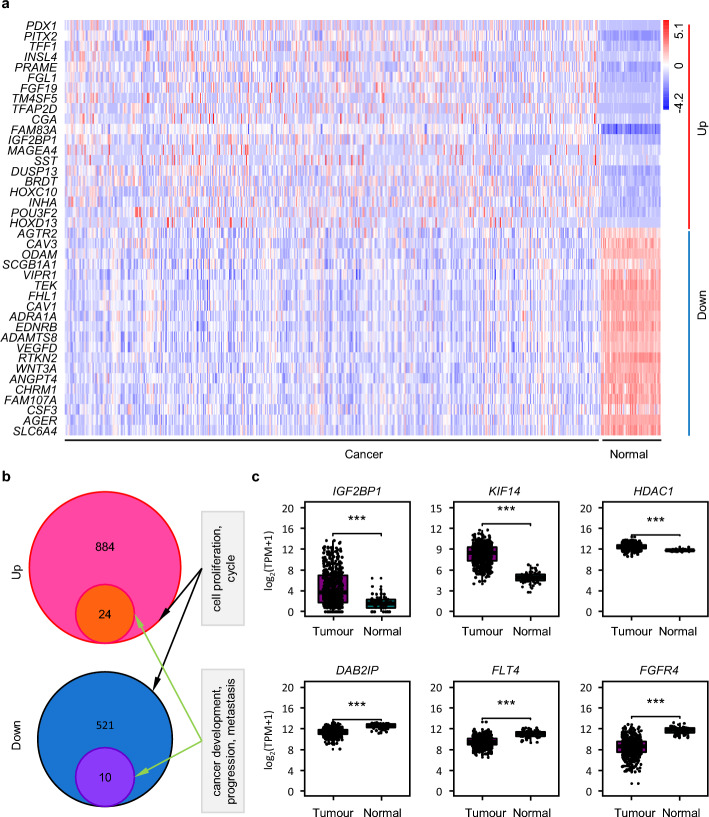
Proliferation-cycle related DEGs in LUAD. **a** Heatmap visualizing transcriptomic profiling of the top 20 proliferation-cycle associated DEGs. **b** Venn diagram illustrating intersection between proliferation-cycle related DEGs and hallmark oncogenic processes (cancer development/progression/metastasis). **c** Boxplot of key proliferation-cycle regulators implicated in oncogenic processes

To elucidate the molecular mechanism of LUAD pathogenesis, we prioritized DEGs linked to cell proliferation (GO:0008283) and cell cycle regulation (GO:0007049) [4, 5]. AmiGO 2 analysis facilitated the identification of 1672 proliferation-cycle related DEGs (Table S2), with the top 40 ranked by expression fold-change visualized in Fig. 3a. Clinical relevance was validated by cross-referencing with UniProt using keywords “cancer development/progression/metastasis,” yielding 24 up-regulated (including *ADGRG1*, *CHEK2*, *ESR1*, *ESRP2*, *FAM98A*, *HDAC1*, *IGF2BP1*, *KCTD13*, *KIF14*, *KISS1*, *KRAS*, *MSH2*, *NAA10*, *NABP2*, *ORC1*, *P2RY6*, *PPME1*, *PROX1*, *PRR5*, *PSRC1*, *PTK6*, *PYGO2*, *UHRF1*, and *VTCN1*) and 10 down-regulated candidates (including *AR*, *DAB2IP*, *FBLN1*, *FGFR4*, *FLT4*, *GPER1*, *ILK*, *MAP3K20*, *NUPR1*, and *VSIR*) (Fig. 3b and c). Consistent with existing literature, these DEGs exhibited pronounced oncogenic activities, exemplified by IGF2BP1, which has been mechanistically linked to non-small cell lung cancer (NSCLC) progression through its regulation of mRNA stability [25]; KIF14, which drives breast cancer cell cycle dysregulation via mitotic spindle assembly modulation [26]; and HDAC1, which facilitates lung cancer metastasis through epigenetic repression of tumor suppressors [27]. Collectively, these findings establish cell proliferation-cycle related DEGs as critical drivers of LUAD pathogenesis, which representing high-priority targets for therapeutic intervention due to their oncogenic roles in diverse malignancies.

**Fig.4:**
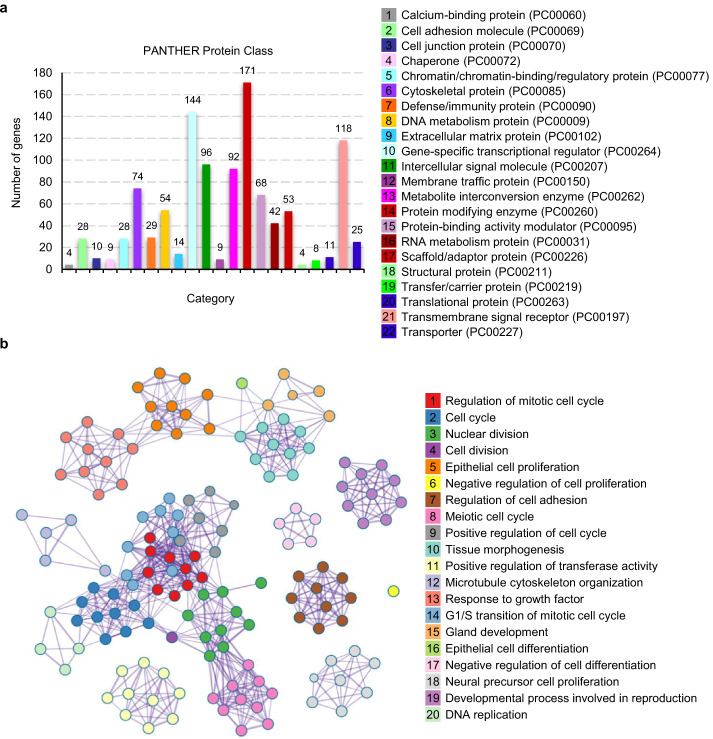
Molecular characterization of 1672 proliferation-cycle related DEGs. **a** PANTHER classification by protein class. **b** Metascape pathway enrichment of top 20 functional modules

PANTHER [12] classification assigned these DEGs to 22 functional categories (Fig. 4a). Over-representation of three gene categories: transcriptional regulation (PC00264, 144 genes, 10.0%), protein modification (PC00260, 171 genes, 11.9%), and transmembrane signal receptors (PC00197, 118 genes, 8.2%) were observed (Fig. 4a). These data implicate signal transduction cascades as pivotal modulators of LUAD cell proliferation, potentially driving oncogenesis through deregulated cellular communication. Pathway mapping identified Wnt (P00057), chemokine and cytokine (P00031), EGF receptor (P00018), p53 (P00059), Cadherin (P00012), and FGF (P00021) signaling as top dysregulated cascades (Table 1). Key molecules implicated in the signal transduction cascades encompass: (1) suppressed receptor FZD5 and activated transcription factor LEF1 in Wnt pathway, (2) up-regulated kinases ATR/CHEK2 and disrupted cell cycle inhibitors CDKN1A/CCNE1/CDK1 in p53 pathway, (3) dysregulated receptors FZD3/5/9, kinases FER, FRK, FYN, ERBB2/3/4 in Cadherin pathway.

**Table 1:** Proliferation-cycle related signaling pathways (≥ 5 gene hits)

Index	Category name (Accession)	Gene hit	Gene hit/total genes (%)
1	Gonadotropin-releasing hormone receptor pathway (P06664)	58	3.90%
2	Angiogenesis (P00005)	47	3.20%
3	Wnt signaling pathway (P00057)	43	2.90%
4	Inflammation mediated by chemokine and cytokine signaling pathway (P00031)	43	2.90%
5	CCKR signaling map (P06959)	39	2.60%
6	EGF receptor signaling pathway (P00018)	29	2.00%
7	Alzheimer disease-presenilin pathway (P00004)	27	1.80%
8	p53 pathway (P00059)	27	1.80%
9	Cadherin signaling pathway (P00012)	27	1.80%
10	FGF signaling pathway (P00021)	25	1.70%
11	Interleukin signaling pathway (P00036)	24	1.60%
12	TGF-beta signaling pathway (P00052)	22	1.50%
13	PDGF signaling pathway (P00047)	22	1.50%
14	Apoptosis signaling pathway (P00006)	19	1.30%
15	Integrin signalling pathway (P00034)	19	1.30%
16	Heterotrimeric G-protein signaling pathway-Gq alpha and Go alpha mediated pathway (P00027)	18	1.20%
17	Heterotrimeric G-protein signaling pathway-Gi alpha and Gs alpha mediated pathway (P00026)	18	1.20%
18	Huntington disease (P00029)	17	1.20%
19	Cytoskeletal regulation by Rho GTPase (P00016)	16	1.10%
20	VEGF signaling pathway (P00056)	15	1.00%
21	p53 pathway feedback loops 2 (P04398)	15	1.00%
22	Ras Pathway (P04393)	15	1.00%
23	Endothelin signaling pathway (P00019)	15	1.00%
24	B cell activation (P00010)	15	1.00%
25	T cell activation (P00053)	14	0.90%
26	Parkinson disease (P00049)	14	0.90%
27	PI3 kinase pathway (P00048)	12	0.80%
28	Alzheimer disease-amyloid secretase pathway (P00003)	10	0.70%
29	Oxidative stress response (P00046)	9	0.60%
30	5HT2 type receptor mediated signaling pathway (P04374)	9	0.60%
31	Muscarinic acetylcholine receptor 1 and 3 signaling pathway (P00042)	8	0.50%
32	Thyrotropin-releasing hormone receptor signaling pathway (P04394)	8	0.50%
33	Cell cycle (P00013)	8	0.50%
34	Toll receptor signaling pathway (P00054)	7	0.50%
35	JAK/STAT signaling pathway (P00038)	7	0.50%
36	Oxytocin receptor mediated signaling pathway (P04391)	7	0.50%
37	Histamine H1 receptor mediated signaling pathway (P04385)	7	0.50%
38	Nicotinic acetylcholine receptor signaling pathway (P00044)	6	0.40%
39	Interferon-gamma signaling pathway (P00035)	6	0.40%
40	Angiotensin II-stimulated signaling through G proteins and beta-arrestin (P05911)	6	0.40%
41	Alpha adrenergic receptor signaling pathway (P00002)	5	0.30%
42	De novo purine biosynthesis (P02738)	5	0.30%
43	Notch signaling pathway (P00045)	5	0.30%
44	Insulin/IGF pathway-protein kinase B signaling cascade (P00033)	5	0.30%
45	p38 MAPK pathway (P05918)	5	0.30%
46	FAS signaling pathway (P00020)	5	0.30%

Moreover, Metascape-based network module analysis [13] enabled the dissection of core oncogenic modules governing proliferation-cycle regulatory dynamics, providing critical insights into oncogenic coordination mechanisms (Fig. 4b). The integrated core network modules exhibit robust correlations with nuclear division, cell adhesion, microtubule cytoskeleton dynamics, enzymatic transferase activity, tissue morphogenesis, and DNA replication machinery (Fig. 4b). These findings unravel novel systems-level signatures of proliferation-cycle dysregulation in LUAD, offering critical insights into oncogenic reprogramming mechanisms.

### DEmiRNAs and clinical relevance

miRNA profiling of 567 TCGA-LUAD samples (521 tumors, 46 normals) identified 268 DEmiRNAs (166 up-regulated and 102 down-regualted, |FC|≥ 1.5, p < 0.05) (Table S3). Among these, 150 DEmiRNAs overlapped with OncomiR database entries [16], including 107 up-regulated and 43 down-regulated candidates (Table S4). Notably, 38 out of 150 DEmiRNAs were dual-associated with both tumorigenesis (stage/grade-linked) and prognosis (survival-linked), highlighting their dual roles in LUAD progression and metastasis (Table 2).

**Table 2:** Differentially expressed miRNAs implicated in tumorigenesis and prognostic outcomes

miRNA	log_2_FC	p-value	p.adj	Pathologic Status	Increased in
hsa-miR-31	4.61	3.78E-33	3.28E-32	M	Deceased
hsa-miR-196b	4.01	4.33E-32	3.68E-31	M	Deceased
hsa-miR-192	3.16	1.65E-22	9.12E-22	Unknown	Unknown
hsa-miR-3189	3.10	4.93E-13	1.68E-12	N	Living
hsa-miR-135b	2.95	8.41E-44	1.32E-42	M	Unknown
hsa-miR-548v	2.52	1.38E-17	5.39E-17	T	Living
hsa-miR-3607	2.44	2.92E-20	1.35E-19	M	Living
hsa-miR-142	2.20	9.81E-36	9.27E-35	N	Living
hsa-miR-187	2.04	7.85E-12	2.55E-11	N	Unknown
hsa-miR-200a	1.98	6.99E-37	7.07E-36	M	Living
hsa-miR-551b	1.91	1.99E-10	5.56E-10	M	Living
hsa-miR-21	1.84	1.07E-53	2.68E-52	T	Deceased
hsa-miR-375	1.81	2.14E-12	7.21E-12	T	Living
hsa-miR-424	1.53	4.88E-19	2.07E-18	Unknown	Unknown
hsa-miR-148a	1.48	2.47E-26	1.87E-25	M	Living
hsa-miR-429	1.45	1.81E-19	7.84E-19	M	Deceased
hsa-miR-628	1.44	5.22E-12	1.71E-11	M	Unknown
hsa-miR-106a	1.35	5.37E-10	1.47E-09	T	Unknown
hsa-miR-188	1.16	2.82E-11	8.63E-11	M	Living
hsa-miR-199b	1.07	1.08E-16	3.98E-16	M	Living
hsa-miR-582	0.97	5.60E-05	1.00E-04	M	Deceased
hsa-miR-381	0.89	1.21E-04	2.12E-04	M	Unknown
hsa-miR-379	0.69	2.49E-03	3.87E-03	T	Unknown
hsa-miR-1468	-0.71	3.07E-05	5.73E-05	T	Living
hsa-miR-99a	-0.89	1.03E-11	3.26E-11	M	Living
hsa-miR-138-1	-0.91	4.72E-04	7.84E-04	N	Unknown
hsa-miR-150	-0.92	1.23E-07	2.79E-07	N	Living
hsa-let-7g	-0.95	2.12E-22	1.16E-21	Unknown	Living
hsa-miR-30d	-0.96	4.44E-11	1.33E-10	Unknown	Living
hsa-miR-584	-1.14	4.18E-08	9.93E-08	T	Deceased
hsa-miR-140	-1.38	6.61E-50	1.34E-48	N	Living
hsa-miR-125a	-1.46	6.96E-43	1.06E-41	M	Living
hsa-miR-145	-1.52	5.47E-26	3.94E-25	M	Living
hsa-miR-1976	-1.96	5.04E-54	1.43E-52	Unknown	Living
hsa-let-7c	-1.96	4.60E-42	6.31E-41	Unknown	Living
hsa-miR-30a	-1.98	4.19E-35	3.79E-34	M	Living
hsa-miR-195	-2.15	3.17E-64	1.23E-62	N	Living
hsa-miR-133b	-2.46	1.08E-23	6.56E-23	M	Living

### miR-31 orchestrates CDK1/E2F2-driven cell cycle progression

A key candidate, miR-31, exhibited strong clinical correlations with pathologic M/T/N status (log₂FC = 4.61 in deceased patients) (Table 2) and was previously linked to lymph node metastasis via PRPF4B/WDR82/NR3C2 regulation [28]. Survival analyses further confirmed its prognostic value, with significant associations to recurrence-free survival (RFS) and overall survival (OS) [29, 30]. These data collectively position miR-31 as a critical mediator of LUAD aggressiveness and a potential biomarker.

Although the significance of other miRNA-mRNA interactions in the progression of LUAD is undeniable, there are substantial grounds to assert that the miR-31/CDK1-E2F2 interaction holds particular importance in this context. This assertion is supported by a range of factors, including not only the log_2_FC values, recurrence-free survival outcome, overall survival outcome, but also PPI network hub analysis, and the tumor-associated biological functions of miRNAs and their target genes that are documented in the literature. Specifically, to investigate the functional interplay between miR-31 and proliferation-cycle associated DEGs, we performed an in-silico analysis using miRWalk2.0, which predicted 260 potential target genes. Gene Ontology (GO) analysis of miR-31 targets revealed significant enrichment of biological processes related to cell cycle and cell proliferation regulation, including mitotic cell cycle process, regulation of cell cycle, cell division, cell cycle phase transition, epithelial cell proliferation and nuclear division. In addition to the cell cycle related pathway, KEGG analysis also revealed enrichment of carcinoma-related pathways, e.g., pathways in cancer, JAK-STAT signaling pathway, melanoma, prostate cancer, pancreatic cancer, chronic myeloid leukemia, microRNAs in cancer, and gastric cancer. These findings not only corroborate the established role of miR-31 as a key regulator of cell proliferation but also extend its functional implications to oncogenesis (uploaded as related files).

**Fig. 5:**
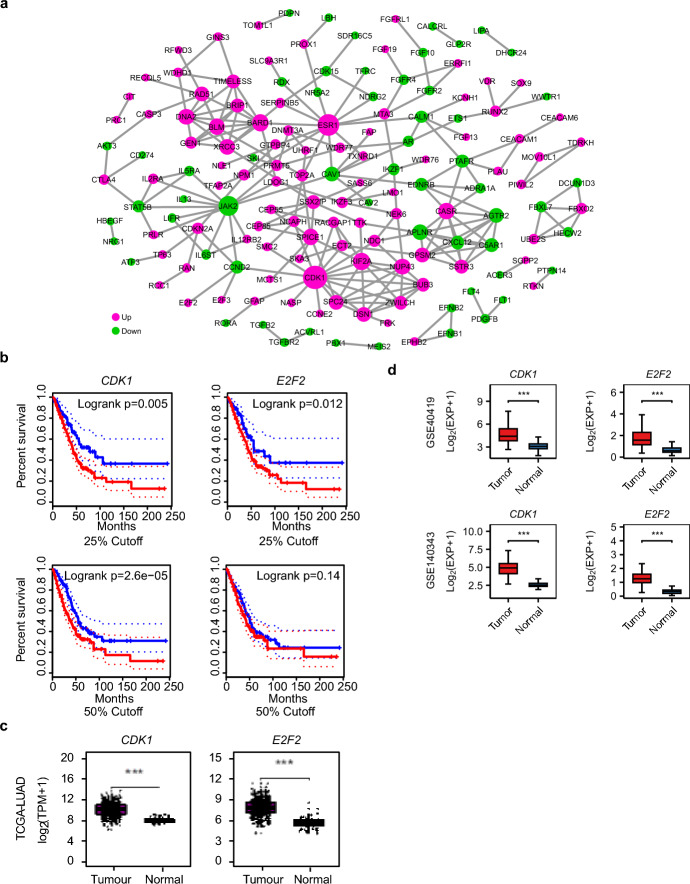
MiR-31 regulatory network targeting CDK1/E2F2 axis. **a** Protein–protein interaction network of miR-31-regulated genes. **b** Kaplan–Meier survival curves stratified by CDK1/E2F2 expression status (high vs. low cohorts). **c** Quantitative assessment revealed significantly elevated CDK1 and E2F2 expression in LUAD specimens compared to adjacent normal tissues. **d** External validation of CDK1 and E2F2 overexpression in LUAD via GEO dataset GSE40419 and GSE140343

The PPI network constructed from these targets identified CDK1 as the central hub, with a remarkably high degree of connectivity, specifically a degree value of 42 (Fig. 5a). CDK1, also known as Cyclin-dependent kinase, forms complexes with various cyclins to orchestrate key events during the cell cycle regulation, including the critical G2/M phase transition, which is essential for cell division. Its extensive interactions with numerous other proteins in the PPI network reflect its crucial position in coordinating multiple cellular processes related to cell cycle and proliferation, thereby making it the central hub of the network (Fig. 5a). Besides, CDK1, along with ESR1, JAK2, KIF2A, BARD1, and CASR, exhibited interactions with more than ten metastasis-associated genes, underscoring their potential roles in cancer progression (Fig. 5a, pink nodes). Kaplan–Meier analysis demonstrated CDK1 overexpression significantly correlated with poor overall survival (Fig. 5b, p = 0.005), consistent with its 5.5-fold up-regulation in LUAD tissues (Fig. 5c and Table S1, p < 0.001). Other crucial regulators, despite low network connectivity, draw our attention owing to marked tumor-normal tissue differences and robust association with cell proliferation. For example, E2F2 exhibited limited network connectivity (Fig. 5b, degree = 1), but existing literatures demonstrate that it directly activates numerous cell cycle related genes via promoter binding [31]. Elevated E2F2 expression similarly predicted poor survival (Fig. 5b, p = 0.012), with 5.1-fold increased expression in LUAD (Fig. 5c, p < 0.001). We also conducted external validation of CDK1 and E2F2 expression using the NCBI GEO LUAD dataset GSE40419 and GSE140343 [14, 15]. Statistical analysis revealed significantly elevated expression of both genes in LUAD tissues compared to normal controls (p < 0.001) (Fig. 5d), consistent with the expression patterns observed in the publicly available TCGA dataset. Notably, the interaction between miR-31 and CDK1 has been implicated in modulating bladder cancer growth [32] and glioma cell proliferation [33]. Research demonstrates that the miR-31/E2F2 interaction functions as a critical regulator across multiple cancers, driving colon cancer proliferation [34] and correlating with poor gastric cancer outcomes through miR-31 suppression [35]. Moreover, miR-31 knockdown induced G2/M cell cycle arrest [36], while miR-31 overexpression disrupted CDK1 activity [37] and E2F2 transcription [35], as confirmed by luciferase reporter assays and western blot experiments. Collectively, this analysis establishes miR-31 as a dual inhibitor of CDK1/E2F2-driven tumor progression, providing a mechanistic basis for its tumor-suppressive role in LUAD, simultaneously highlighting the promising therapeutic potential of CDK1 and E2F2 as a LUAD targets.

### Potential prioritized drugs targeting CDK1-mediated oncogenic pathways

**Fig. 6:**
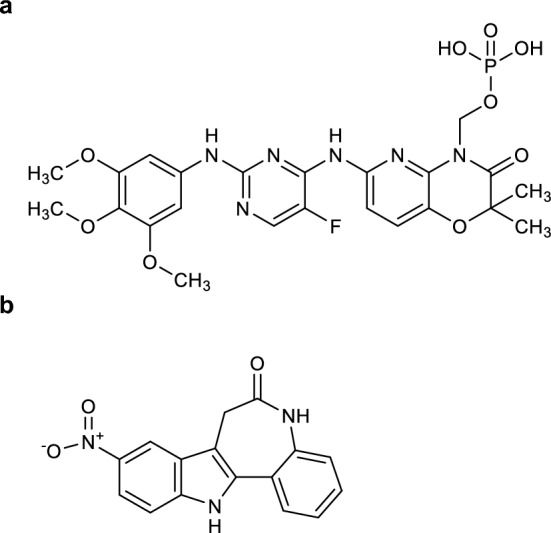
Chemical structures of CDK1-targeting drug candidates. **a** Fostamatinib. **b** Alsterpaullone

The Connectivity Map [23] analysis was employed to identify therapeutic drugs capable of reversing perturbations induced by aberrant expression of miR-31 targets CDK1 and E2F2. This analysis identified two CDK1 inhibitors with probable efficacy: fostamatinib (Fig. 6a) and alsterpaullone (Fig. 6b). Fostamatinib, a FDA-approved drug [38], demonstrated anti-angiogenic and anti-proliferative effects in NSCLC and hematological malignancies via blocking B-cell receptor signaling, consistent with its mechanism of action in chronic lymphocytic leukemia [39, 40]. Recent studies showed that fostaminib may act as a CDK1 inhibitor [41, 42], consistent with our anticipation. However, its direct inhibitory effect in LUAD remains unverified and requires further experimental investigation. Alsterpaullone, an experimental validated CDK1 inhibitor [43, 44], showed Group 3 medulloblastoma growth suppression in vitro, warranting LUAD-specific validation. No direct inhibitors targeting E2F2 were identified, revealing a critical gap in therapeutic strategies against transcription factor-driven oncogenesis. These findings highlight the translational potential of CDK1 inhibitors in LUAD therapeutics.

CDK1 inhibitors represent a distinct class of agents with a specific focus on cell cycle regulation. To effectively showcase the unique advantages and potential of CDK1 inhibitors (including fostamatinib and alsterpaullone) in the treatment of LUAD, it is necessary to quantify and contrast their therapeutic effects across diverse cancer contexts. However, publicly available clinical information specifically regarding fostamatinib's (or alsterpaullone’s) efficacy in LUAD is scarce or lacking, particularly concerning response rates, progression-free survival, and overall survival. This scarcity points to the need for further clinical trials specifically designed to evaluate the efficacy of CDK1 inhibitors in LUAD. Future clinical trials should strengthen the rationale for prioritizing CDK1 inhibitors in the management of LUAD, offering new therapeutic avenues for patients with this challenging malignancy.

## Discussion

**Fig. 7:**
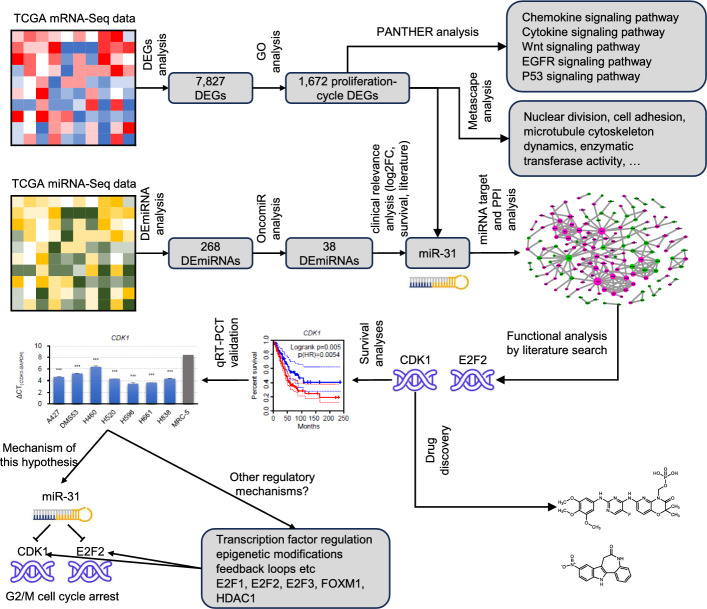
Graphical depiction of key findings in the article

Aberrant cell proliferation and cell cycle deregulation are a defining feature of LUAD pathogenesis, orchestrating uncontrolled tumor initiation, aggressive progression, metastatic dissemination, and disease recurrence [4, 5]. In this study, we identified 1672 proliferation-cycle associated DEGs and 38 oncogenic DEmiRNAs implicated in the pathogenesis of LUAD. Additionally, our research revealed that miR-31 plays a pivotal role in mediating cell cycle arrest by targeting CDK1 and E2F2 genes, thereby inhibiting LUAD cell proliferation. These findings not only provide a new perspective for understanding the pathogenesis of LUAD, but also establish miR-31 as a novel biomarker for assessing the proliferative potential of LUAD. Furthermore, our study delineated the specific mechanistic actions of the miR-31/CDK1/E2F2 network in LUAD and proved the feasibility of CDK1 and E2F2 as potential therapeutic targets. The key findings outlined in this article were depicted in Fig. 7, offering a straightforward view of the research outcomes.

Specifically, we identified 1672 proliferation-cycle associated DEGs, with PANTHER analysis revealing significant enrichment of transmembrane receptors, kinases, and TFs. Key oncogenic pathways including chemokine/cytokine, Wnt, EGFR, and p53 signaling emerged as central regulators, consistent with their established roles in LUAD progression [45–49]. This finding provides critical mechanistic insights with actionable therapeutic potential. Inhibitors of these signaling pathways show promise in reversing immunosuppressive tumor microenvironments. By depleting cancer stem cells and enhancing tumor sensitivity to conventional therapies, these agents represent a transformative strategy for overcoming therapeutic resistance in proliferation-cycle genes-driven malignancies.

Differential miRNA expression profiling identified 38 DEmiRNAs (Table 2), including let-7, miR-21, and miR-31, previously implicated in cancer proliferation [4, 50–53]. The robust correlation between miR-31 overexpression and poor survival outcomes implies its clinical significance, warranting exploration of its potential as a prognostic biomarker in LUAD management. Mechanistically, miR-31 directly targeted CDK1 and E2F2 (Fig. 5a), master regulators of G2/M transition and S-phase entry, respectively. Elevated CDK1 or E2F2 expression exhibited no survival advantage, validating their oncogenic roles (Fig. 5b and c). It is noteworthy that we simultaneously observed elevated expression levels of miR-31, CDK1, and E2F2 in LUAD. While miR-31 typically inhibits its target genes, this seemingly contradictory finding may indicate the presence of additional regulatory mechanisms, such as transcription factor-mediated up-regulation, contributing to the overexpression of CDK1 and E2F2. This stresses the immense complexity of tumor regulation. These findings unravel a critical regulatory axis in LUAD progression, wherein miR-31 emerges as a pivotal tumor suppressor by suppressing oncogenic genes CDK1 and E2F2. Collectively, these insights not only enhance our mechanistic understanding of miRNA-driven cancer progression but also suggest miR-31/CDK1-E2F2 axis as a promising therapeutic target for disrupting oncogenic signaling cascades in LUAD.

The miR-31/CDK1 axis represents a novel therapeutic target. Our drug screening prioritized fostamatinib and alsterpaullone, two CDK1 inhibitors demonstrating dose-dependent antiproliferative activity. These compounds effectively reversed miR-31-mediated cell cycle dysregulation, specifically alleviating G2/M phase arrest. Clinical translation is further supported by fostamatinib's FDA approval for hematological malignancies, warranting LUAD repurposing trials. Although the clinical relevance of the miR-31/CDK1 axis requires further validation, our findings collectively suggest it as an actionable therapeutic target with clinical translation potential.

The diagnostic and therapeutic implications of miR-31 in LUAD are substantial. Studies have demonstrated that miR-31 shows promise as a tumor serum biomarker across multiple non-LUAD malignancies. In neck squamous cell carcinoma, serum miR-31 levels are markedly increased compared to normal controls and can distinguish patients from controls, also correlating with poor prognosis [54]. Additionally, miR-31 is significantly elevated in the serum of EBV-associated oropharyngeal cancer patients [55], and in colorectal cancer, miR-31 is among dysregulated serum miRNAs with diagnostic potential [56]. Although there is a lack of direct evidence to support the clinical application of miR-31 as a serum biomarker for LUAD, its high expression in LUAD tissues and the serum correlation observed in other tumor types suggest its potential application value as a non-invasive indicator for early LUAD detection and prognosis monitoring. Targeting the miR-31/CDK1-E2F2 axis offers a promising therapeutic strategy. This can be achieved by either (1) restoring miR-31 levels to directly suppress CDK1 and E2F2 expression, thereby inducing G1/S phase cell cycle arrest, or (2) using selective inhibitors against CDK1 and E2F2 to block their downstream proliferative signaling. Such dual approaches could synergistically enhance antitumor efficacy while minimizing resistance, providing novel avenues to complement existing chemotherapy or targeted therapies in precision oncology.

Despite these achievements, there are still some aspects that need to be improved. Firstly, corresponding experimental validations, such as luciferase reporter assays, in vivo animal model studies, and clinical trials, are still need to further verify the precise mechanism of action of the miR-31/CDK1-E2F2 network and the candidate drugs in LUAD. Secondly, additional regulatory mechanisms of CDK1 and E2F2 should be explored. We simultaneously observed elevated expression levels of miR-31, CDK1, and E2F2 in LUAD. This appears to contradict the conclusion that miR-31 acts as a suppressor gene for CDK1 and E2F2. The possible causes mainly fall into two aspects. In complex biological systems, numerous factors can affect gene expression, and the observed simultaneous high expression of these genes might result from other regulatory mechanisms or feedback loops. Firstly, the expression of genes like CDK1 and E2F2 is not solely controlled by miR-31. There is numerous other transcription factors, epigenetic modifications, and non-coding RNAs that can influence their transcription. For example, certain oncogenic transcription factors and/or epigenetic modification factors might be highly active in the LUAD cells, directly promoting the transcription of CDK1 and E2F2. We observed that E2F1, E2F2, E2F3, FOXM1, and HDAC1 exhibit significantly elevated expression levels in tumor tissues (Table S1). Notably, these proteins collectively function as upstream regulators capable of activating the expression of downstream targets CDK1 and E2F2 [57–59]. Chromatin immunoprecipitation assays confirmed direct binding of FOXM1 to the promoter regions of E2F1, E2F2, and CDK1 [58]. The histone deacetylases HDAC1 and HDAC2 play a critical role in maintaining the enzymatic activity of the cell cycle kinase CDK1, thereby facilitating G2/M phase progression [59]. These transcription factors and/or epigenetic modification factors could override the suppressive effect of miR-31 on these genes, leading to their increased expression despite the presence of high levels of miR-31. Additionally, in biological systems, feedback loops and compensatory mechanisms are common. When miR-31 suppresses CDK1 and E2F2, the cells might activate compensatory mechanisms to restore the expression of these oncogenic genes. For instance, the reduced levels of CDK1 and E2F2 due to miR-31 action could trigger a signaling cascade that up-regulates the transcription of these genes through alternative pathways. This feedback loop could result in the observed simultaneous elevation of miR-31, CDK1, and E2F2 levels. Thirdly, efforts will be directed towards the development of innovative therapeutic agents specifically targeting the miR-31/CDK1-E2F2 network, which could yield new LUAD treatment options.

In summary, this study establishes miR-31 as a pivotal indicator associated with LUAD pathogenesis, demonstrating its dual modulatory effects on CDK1 and E2F2 through 3'-UTR binding. However, its diagnostic performance for LUAD requires multi-center validation in independent cohorts. The prioritized therapeutic inhibitors, represent potential strategies for disrupting the miR-31/CDK1 axis, but still require comprehensive preclinical evaluation.

## Limitations

Although our study has shed light on the role of miR-31 in suppressing LUAD cell proliferation through CDK1 and E2F2-mediated cell cycle arrest and offers promising avenues for future therapeutic interventions, it is crucial to recognize certain limitations that may impact the interpretation and generalizability of our findings. These limitations mainly include: (1) Clinical metadata limitations: the clinical metadata limitations within TCGA dataset, particularly the incomplete treatment history, can significantly affect prognostic interpretations. Such interpretations often rely on long-term follow-up data to assess the durability of treatment responses and the likelihood of late-onset complications. However, the lack of comprehensive treatment history can make it challenging to accurately track the long-term outcomes of LUAD patients. (2) Lack of In Vivo Validation: There is a lack of extensive in vivo validation studies to further confirm the therapeutic potential of targeting the miR-31/CDK1-E2F2 network in LUAD. (3) limitations in drug discovery: CMAP's reliance on cell line models and static transcriptional signatures may fail to capture intratumoral heterogeneity and context-dependent regulatory dynamics. This methodological constraint increases the likelihood of spurious drug-LUAD proliferation associations, particularly when extrapolating to complex in vivo systems. In summary, while our study offers valuable insights, these limitations should be considered when interpreting the results and designing future research endeavors.

## Supplementary Information

Supplementary Material 1: Table S1. DEGs identified in human LUAD compared to adjacent lung normal cells.

Supplementary Material 2: Table S2. DEGs associated with cell proliferation and cell cycle regulation in LUAD vs. normal lung cells.

Supplementary Material 3: Table S3. DEmiRNAs in LUAD vs. normal lung cells

Supplementary Material 4: Table S4. DEmiRNAs implicated in LUAD pathogenesis and progression

## Data Availability

Raw RNA-Seq read count data analyzed were downloaded from The Cancer Genome Atlas (TCGA, https://portal.gdc.cancer.gov). Other data analyzed during this study are provided as supplementary information.
